# Significance of Time Until PSA Recurrence After Radical Prostatectomy Without Neo- or Adjuvant Treatment to Clinical Progression and Cancer-Related Death in High-Risk Prostate Cancer Patients

**DOI:** 10.3389/fonc.2019.01286

**Published:** 2019-11-22

**Authors:** Zilvinas Venclovas, Mindaugas Jievaltas, Daimantas Milonas

**Affiliations:** Department of Urology, Lithuanian University of Health Sciences, Medical Academy, Kaunas, Lithuania

**Keywords:** prostate cancer, high-risk, locally advanced, biochemical recurrence, radical prostatectomy, PSA persistence

## Abstract

**Objective:** The aim of our study was to evaluate the impact of time until biochemical recurrence (BCR) after radical prostatectomy (RP) without neo- or adjuvant treatment on clinical progression (CP) and cancer-related death (CRD) in high-risk prostate cancer (HRPCa) patients.

**Materials and methods:** A total of 433 men with clinically HRPCa treated between 2001 and 2017 were identified. HRPCa was defined as clinical stage ≥T2c and/or biopsy Gleason score (GS) ≥8 and/or preoperative prostate specific antigen (PSA) value ≥20 ng/ml. Exclusion criteria were neo- or adjuvant treatment and incomplete pathological or follow-up data. BCR was defined as two consecutive PSA values ≥0.2 ng/ml after RP. CP was identified as skeletal lesions, local or loco-regional recurrence. CRD was defined as death from PCa. All men were divided into two groups according to BCR. The chi-square and *t*-tests were used to compare baseline characteristics between groups. Biochemical progression free survival (BPFS), clinical progression free survival (CPFS), and cancer-specific survival (CSS) rates were estimated using Kaplan–Meier analysis. Patients with detected BCR were analyzed for prediction of CP and CRD with respect to time until BCR. The impact of baseline parameters on BCR, CP, and CRD was assessed by Cox regression analysis.

**Results:** BCR, CP, and CRD rates were 47.8% (207/433), 11.3% (49/433), and 5.5% (24/433), respectively. Median (quartiles) time of follow-up after RP was 64 (40–110) months. Ten-year BPFS rate was 34.2%; CPFS, 81%; and CSS, 90.1%. Men with detected BCR were analyzed for prediction of CP and CRD with respect to time until BCR. The most informative cutoff for time from RP until CP and CRD was ≤ 1 year (*p* < 0.008). According to this cutoff, men were divided into two groups: BCR detected within 1 year and after a 1-year period. Ten-year CPFS was 49.8% in men with early BCR vs. 81.1% in men with late BCR; CSS was 70.9 vs. 92.8% (*p* = 0.001). Multivariable analysis confirmed that time until BCR within 1 year predicts CP (*p* = 0.005) and CRD (*p* = 0.03).

**Conclusions:** Early BCR is associated with poorer oncological outcomes. The presented results may help both to improve follow-up strategy and opt for more aggressive multimodal treatment of HRPCa in men with very early BCR.

## Introduction

Prostate cancer (PCa) remains one of the most often diagnosed cancers among men. There were 1.3 million new cases of cancer in 2018 worldwide (1). According to the D'Amico classification, while the proportion of high-risk prostate cancer (HRPCa) has decreased due to the prostate-specific antigen (PSA) era, 1 out of 3 patients will still be diagnosed as having high-risk disease features (2). The optimal treatment for HRPCa remains debatable because of the lack of randomized clinical trials (3–6). A recently published study showed similar oncological outcomes of RP with external beam radiotherapy and low-dose-rate brachytherapy (7).

Although surgical treatment provides adequate disease control for all risk localized PCa, 1/4 of patients might experience a disease recurrence (2, 8). The most common test used to analyze disease recurrence is a detectable PSA concentration rate in the postoperative period. Although there is an official follow-up strategy after RP, it has its own limitations because it was created for all PCa risk groups. According to the European Association of Urology (EAU) guidelines, biochemical recurrence (BCR) is diagnosed after two consecutive PSA ≥ 0.2 ng/ml following RP (9). Special attention should be paid to patients with HRPCa features: they experience BCR more often as the 10-year BCR rate may increase to 85% (6, 10). Furthermore, patients that have a disease recurrence also have an increased risk of developing clinical progression (CP) and of achieving higher cancer-specific and overall mortality rates (11, 12). The timing of BCR is essential; early recurrence is associated with poorer oncological outcomes (11, 13).

Up till now, there have only been a handful of studies where time to BCR and its effect on survival have been analyzed for patients with HRPCa treated with RP without neo- or adjuvant therapy (13–18). In most studies, HRPCa represents only a small number of patients, whereas cases with low and intermediate PCa make up the bulk.

The aim of our study was to evaluate the impact of time until BCR on CP and cancer-related death (CRD) in HRPCa patients that were treated with RP without neo- or adjuvant treatment.

## Materials and Methods

### Patient Population

Between 2001 January and 2017 December, 2387 men with clinically localized PCa underwent open radical prostatectomy at the Department of Urology of Lithuanian University of Health Sciences. Preoperative data included age, clinical stage (cT), preoperative PSA, biopsy Gleason score (GS), and percentage of positive biopsy cores. HRPCa was defined using D'Amico criteria: ≥ T2c and/or biopsy GS ≥ 8 and/or preoperative PSA value ≥ 20 ng/ml (19). Of all the men who underwent RP, 469 met HRPCa criteria and were included into the study. Pathological stage (pT), surgical margins status (R), pathological GS, number of lymph nodes removed, and number of positive lymph nodes were registered after RP. Pathological stage was assessed using the 2002 TNM system, and tumor grading was classified by using the revised 2005 Gleason grading system (20) and 2014 ISUP suggested grade grouping (21).

PSA measurement after surgery was recommended at first, third, and every 3 months of the first year, biannually in the second and third year, and annually thereafter. First PSA value ≥0.1 ng/ml after RP within 6 and 8 weeks was defined as persistent. PSA dynamics and additional treatment were registered in these cases. BCR was defined as two consecutive PSA values ≥0.2 ng/ml. Adjuvant therapy was defined as androgen deprivation therapy (ADT) or radiation therapy (RT) or both (ADT + RT) within 6 months after RP when post-operative PSA value was <0.2 ng/ml. Salvage therapy was defined as RT or ADT or RT + ADT or salvage lymph nodes dissection (LND) after detected BCR or when persistent PSA was ≥0.2 ng/ml. Time from RP to any kind of additional treatment was registered.

CP was identified when skeletal or visceral lesions were confirmed by bone scan, computer tomography (CT), positron emission tomography (PET/CT), or magnetic resonance imaging (MRI); local or loco-regional recurrence was confirmed by biopsy or salvage surgery. Time from BCR to CP was recorded. CRD was defined as death from PCa. Biochemical progression free survival (BPFS) was defined as the time from the operation to the day of BCR, clinical progression free survival (CPFS) was defined as the time from the operation to the day of CP, and cancer-specific survival (CSS) was defined as the time from the operation to the day of death from PCa.

Exclusion criteria were neo- or adjuvant treatment and incomplete pathological or follow-up data. Thirty-seven men were excluded from the study.

### Statistics

All men were divided into two groups according to BCR. Medians, interquartile ranges, and frequencies were used for descriptive statistics. The chi-square and *t*-tests were used to compare pre- and postoperative characteristics between the following groups: age, PSA, cT, biopsy GS, percentage of positive cores, number of risk factors according to D'Amico classification, pathological GS, pT, pelvic lymphonodectomy (PLND), lymph node invasion (LNI), and R1.

BPFS, CPFS, and CSS rates were estimated using Kaplan–Meier analysis.

The patients with detected BCR were analyzed for the prediction of CP and CRD with respect to time until BCR (≤1, 1–2, 2–3, 3–4, and 4–5 years). Patients with persistent PSA were not excluded from the initial analysis. Additionally, a sub-analysis was performed to evaluate the impact of persistent PSA on CP and CRD.

Age, preoperative PSA, cT, biopsy GS, percentage of positive cores, number of risk factors according to D'Amico classification, pathological GS, pT, LNI, and R1 were evaluated for BCR, CP, and CRD in the univariable analysis. Only significant covariates were used in the multivariable analysis by using Cox regression backward conditional stepwise method. The number of risk factors by D'Amico classification was excluded from the explanatory variables for correlation in the multivariable analysis because PSA, cT, and pathological GS influence the number of risk factors by D'Amico classification.

All analyses were performed using the SPSS software (version 23.0, SPSS). A *p*-value of < 0.05 was considered statistically significant. The Lithuanian University of Health Sciences Ethical Committee approved prospective collection of the data (BE-2-48). All patients signed a consent form provided before RP.

## Results

### Baseline Characteristics

The total number of participants included in the final analysis was 433 men. Table 1 summarizes the characteristics of our study cohort. The median patient age at the time of RP was 65 years old (IQR 60–68). According to the D'Amico risk classification for PCa, 298 men (68.8%) had one risk factor. Most of the patients had ≥ cT2c (*n* = 323, 74.6%) and most common biopsy GS was 6 (3 + 3) (*n* = 142, 32.8%). However, after RP, pathological GS 7 (3 + 4) was the most frequent (*n* = 173, 40%) and almost half of the patients had pT3a (*n* = 194, 44.8%).

**Table 1:** Patient characteristics.

**Parameter**	**No BCR (226)**	**BCR (207)**	***p*-value**	**All (*n* = 433)**
Age (years): median (IQR)	65 (60–68)	65 (59–69)	0.68	65 (60–68)
PSA (ng/ml): *n* (%)
<20	185 (81.86)	146 (70.5)	0.006	331(76.4)
≥20	41 (18.1)	61 (29.5)		102 (23.6)
Clinical stage: *n* (%)
cT1–cT2a	35 (15.5)	33(15.9)	0.96	68 (15.7)
cT2b	25 (11.1)	17 (8.2)		42 (9.7)
≥cT2c	166 (73.4)	157 (75.8)		323 (74.6)
Biopsy GS: *n* (%)
6	92 (40.7)	50 (24.2)	<0.0001	142 (32.8)
3 + 4	64 (28.3)	55 (26.6)		119 (27.5)
4 + 3	12 (5.3)	19 (9.2)		31 (7.2)
8	46 (20.4)	49 (23.7)		95 (21.9)
9 – 10	12 (5.3)	34 (16.4)		46 (10.6)
% of positive cores: median (IQR)	38 (25–62)	50 (33–75)	<0.0001	50 (29.25–67)
D'Amico HRPCa: *n* (%)
1 risk factor	178 (78.8)	120 (58)	<0.0001	298 (68.8)
2 risk factors	47 (20.8)	74 (35.7)		121 (27.9)
3 risk factors	1 (0.4)	13 (6.3)		14 (3.3)
Pathological GS: *n* (%)
6	33 (14.6)	11 (5.3)	<0.0001	44 (10.2)
3 + 4	122 (54.0)	51 (24.6)		173 (40)
4 + 3	31 (13.7)	36 (17.4)		67 (15.5)
8	24 (10.6)	31 (15)		55 (12.7)
9 – 10	16 (7.1)	78 (37.7)		94 (21.7)
Pathologic stage: *n* (%)
pT2	104 (46)	34 (16.4)	<0.0001	138 (31.9)
pT3a	110 (48.7)	84 (40.6)		194 (44.8)
**≥**pT3b	12 (5.3)	89 (43)		101 (23.3)
PLND: *n* (%)	142 (62.8)	181 (87.4)	<0.0001	323 (74.6)
LNI: *n* (%)	1 (0.4)	55 (30.4)	<0.0001	56 (17.4)
R1: *n* (%)	66 (30.6)	122 (58.9)	<0.0001	188 (43.4)
Rx	10(4.4)	10 (4.8)		20 (4.6)

*IQR, interquartile range*.

Eighty-seven patients (61.27%) with cT1–cT2 were upstaged after the surgery to ≥ pT3. However, 77 patients (27.3%) were downstaged from cT3 to pT2.

Ninety-one (64.1%) tumors graded GS 6 at biopsy were upgraded to GS 7 and 10 patients (7%) were upgraded up to GS ≥ 8 after the surgery. Thirty-seven men (24.7%) with biopsy GS 7 were upgraded to GS ≥ 8; however, 1 patient (0.7%) was downgraded to GS 6. Thirty-seven patients (26.3%) with biopsy GS ≥ 8 were downgraded to GS 7 after RP.

Of 433 men, 323 (74.6%) underwent PLND; median 7 (IQR 5–12) lymph nodes were removed. LNI was found in 56 patients (17.4%) with a median of 2 (IQR 1–3) positive lymph nodes. In 40 cases (71.5%), one or two positive nodes were detected, while in 16 (28.5%) cases, three and more were detected.

When patients were stratified according to the biochemical relapse, there were significant difference between preoperative PSA, biopsy GS, percentage of positive biopsy cores, number of D'Amico risk factors, pathological GS, PLND, LNI and R1 (from *p* = 0.006 to *p* < 0.0001) (Table 1).

### The Frequency of BCR, CP, CRD, and Rates of BPFS, CPFS, CSS

**Figure 1:**
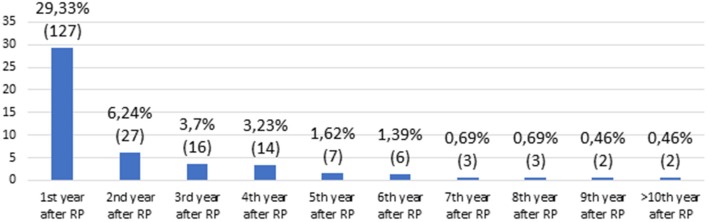
Risk of biochemical recurrence by the following year after radical prostatectomy (%).

Median time of follow-up after RP was 64 (IQR 40–110) months. Over this time, 207 men (47.8%) experienced BCR. One hundred twenty-seven men (61.35%) had BCR in the following year after RP, 27 (13.04%) in the second year, 16 (7.73%) in the third, 14 (6.76%) in the fourth, 7 (3.38%) in the fifth, and 16 (7.73%) patients had BCR after 5 years (Figure 1). Of 207 men, 181 (87.44%) received salvage radiotherapy (sRT) or hormone therapy (HT) or both sRT + HT due to BCR.

CP was diagnosed in 49 (11.3%) cases. Median time from BCR to CP was 17 (IQR 9.5–35) months. Twelve men (24.5%) had metastases in lymph nodes, 11 (22.4%) had metastases in bones, 19 (36.8%) had metastases in lymph nodes and bones, 1 (2%) had visceral metastases, and 6 (12.2%) had local recurrence in the surgical bed. During the follow-up, 72 patients (16.6%) died. In 24 cases (5.5%) PCa was the cause of death.

According to the D'Amico risk classification, the 5-year BPFS rate after RP of patients with one risk factor was 57.7%, and that with two factors was 34.4%. All patients with three risk factors had BCR in the first 5 years after RP (*p* < 0.0001) (Supplementary Figure 1).

In all study cohorts, 5- and 10-year BPFS rate was 49.2 and 34.2%, respectively. CPFS rate was 89.2 and 81% and CSS rate was 95.6 and 90.1%, respectively.

### Uni- and Multivariable Regression Analyses Predicting BCR

Preoperative features were analyzed to determine which factors significantly predict BCR after RP. In the univariable analysis, PSA, biopsy GS, percentage of positive biopsy cores, and number of D'Amico risk factors were significant (from *p* = 0.007 to *p* < 0.0001). These factors were used in the multivariable analysis and showed that higher grade of biopsy GS and level of preoperative PSA are the most informative predictors for BCR (from *p* = 0.006 to *p* < 0.0001) (Table 2).

**Table 2:** Univariable and multivariable logistic regression analyses while using preoperative factors for predicting BCR.

	**Univariable analysis**	**Multivariable analysis**
**Predictors**	**Odds ratio (95% CI)**	***p*** **value**	**Odds ratio (95% CI)**	***p*** **value**
Age	0.99 (0.97–1.02)	0.6	–	–
PSA ng/ml
<10	Reference	0.003	Reference	0.006
10–20	1.65 (1.9–2.28)	<0.0001	1.64 (1.15–2.34)	<0.0001
>20	1.84 (1.31–2.57)		2.02 (1.39–2.95)	
Clinical stage
T1–T2a	Reference	0.47	–	–
T2b	0.8 (0.45–1.45)	0.66		
≥T2c	1.09 (0.75–1.59)			
Biopsy GS
6	Reference	0.007	Reference	0.019
3+4	1.7 (1.15–2.5)	<0.0001	1.7 (1.09–2.68)	0.004
4+3	3.03 (1.77–5.18)	0.005	2.42 (1.33–4.41)	0.001
8	1.77 (1.19–2.63)	<0.0001	2.19 (1.38–3.47)	<0.0001
9–10	3.55 (2.28–5.53)		3.84 (2.31–6.38)	
No. of risk factors
1 risk factor	Reference	<0.0001	–	–
2 risk factors	2.01 (1.5–2.69)	<0.0001		
3 risk factors	3.58 (2.01–6.38)			
% of positive cores	1.01 (1.01–1.02)	<0.0001	1.01 (1–1.02)	0.001

*CI, confidence interval*.

In the univariable analysis, all postoperative factors predict BCR after RP (*p* < 0.0001). These factors were used in the multivariable analysis and showed that higher grade of pathological GS, pT, LNI, and R1 are the most informative predictors for BCR (from *p* = 0.028 to *p* = 0.005) (Table 3).

**Table 3:** Univariable and multivariable logistic regression analyses, using post-operation features for predicting the presence of BCR.

	**Univariable analysis**	**Multivariable analysis**
**Predictors**	**Odds ratio (95% CI)**	***p*****-value**	**Odds ratio (95% CI)**	***p*****-value**
Pathological GS
6	Reference	0.22	Reference	0.76
3 + 4	1.51 (0.78–2.91)	<0.0001	1.13 (0.51–2.48)	0.072
4 + 3	3.78 (1.9–7.55)	<0.0001	2.13 (0.94–4.84)	0.071
8	3.71 (1.85–7.43)	<0.0001	2.16 (0.94–5.96)	0.005
9 – 10	9.67 (5.01–18.66)		3.15 (1.41–7.0)	
Pathological stage
T2	Reference	<0.0001	Reference	0.021
T3a	2.17 (1.45–3.24	<0.0001	1.78 (1.09–2.91)	0.007
≥T3b	7.2 (4.81–10.77)		2.16 (1.24–3.75)	
LNI	5.42 (3.85–7.63)	<0.0001	1.61 (1.05–2.47)	0.028
R1	2.48 (1.86–3.32)	<0.0001	1.54 (1.1–2.18)	0.014

### Early vs. Late BCR

Patients with detected BCR (*n* = 207) were analyzed for prediction of CP and CRD with respect to time until BCR (≤1, 1–2, 2–3, 3–4, and 4–5 years). The most informative cutoff was BCR in the following year after RP (*p* < 0.008) (Table 4).

**Table 4:** CP and CRD rates according to time of BCR.

**Time of BCR**	**CP, *n* = 49 (%)**	***p***	**CRD, *n* = 24 (%)**	***p***
≤1 year	38 (77.56)	0.008	21 (87.5)	0.005
1–2 years	5 (10.2)	0.5	2 (8.3)	0.47
2–3 years	2 (4.08)	0.27	–	–
3–4 years	1 (2.04)	0.13	1 (4.2)	0.59
4–5 years	1 (2.04)	0.55	–	–

**Figure 2:**
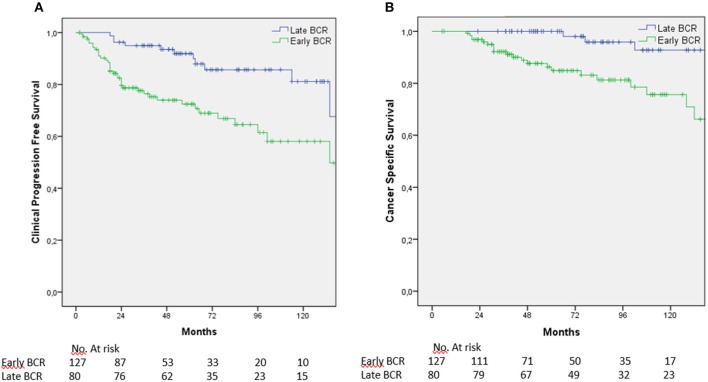
**(A)** CPFS and **(B)** CSS according to BCR time.

According to this cutoff, patients were divided into two groups: BCR detected within 1 year (early BCR) (*n* = 127, 61.4%) and after 1 year (late BCR) (*n* = 80, 38.6%). Five-year and 10-year CPFS was 70.7 and 49.8% in men with early BCR vs. 89.9 and 81.1% in men with late BCR (*p* = 0.001) (Figure 2A); CSS was 84.8 and 70.9% vs. 98% and 92.8% (*p* = 0.001), respectively (Figure 2B).

#### Sub Analysis of PSA Persistence

A total of 130 patients (30%) had PSA persistence (PSA ≥ 0.1 ng/ml). Seventy-three of them (56.2%) had PSA ≥ 0.2 ng/ml and were assigned to early BCR group. Fifty-seven patients (43.8%) had PSA between 0.1 and 0.2 ng/ml and 44 of them (77.2%) harbored BCR during the study period.

A further analysis was done to evaluate whether PSA persistence could be a predictive factor for CP and CRD. In the univariable analysis, PSA persistence was significant for CP (HR: 7.5; *p* < 0.0001) and CRD (HR: 3.7; *p* = 0.004), but it was not meaningful in the multivariable regression analysis (HR: 1.01; *p* = 0.89 and HR: 1.5; *p* = 0.51) when time to BCR (early BCR vs. late BCR) was added (the data are shown in Supplementary Table 1). Therefore, PSA persistence was not included for further CP and CRD analysis as a predictor in order to avoid mismatching PSA follow-up data.

### Uni- and Multivariable Regression Analyses Predicting CP and CRD

Patients with BCR were further analyzed. In the univariable analysis for CP, we found that biopsy GS, number of D'Amico risk factors, percentage of positive biopsy cores, pathological GS, pT, LNI, R1, and early BCR were significant and were used in the multivariable analysis. Only stage pT3b and early BCR were detected as significant prognosticators of CP (*p* = 0.041 and *p* = 0.005, respectively).

In the univariable analysis for CRD, only LNI, R1, and early BCR were significant and were used in the multivariable analysis. All of them correlated with CRD (from *p* = 0.03 to *p* = 0.002) (Table 5).

**Table 5:** Multivariable logistic regression analyses predicting the presence of CP and CRD.

	**CP Multivariable analysis**	**CRD Multivariable analysis**
**Predictors**	**Odds ratio (95% CI)**	***p*****-value**	**Odds ratio (95% CI)**	***p*****-value**
After RP GS
6	Reference		–	–
3 + 4 vs. 6	0.24 (0.04–1.34)	0.1		
4 + 3 vs. 6	0.24 (0.04–1.58)	0.14		
8 vs. 6	0.68 (0.13–3.6)	0.65		
9 – 10 vs. 6	1.99 (0.38–10.43)	0.42		
Pathological stage
T2	Reference	0.69	–	–
T3a vs. T2	1.29 (0.38–4.4)	0.041		
≥T3b vs. T2	3.44 (1.05–11.24)			
LNI	0.89 (0.79–3.65)	0.73	4.1 (1.66–10.14)	0.002
R1	1.7 (0.79–3.65)	0.18	4.2 (1.23–14.38)	0.02
Early vs. late BCR	2.72 (1.35–5.5)	0.005	3.98 (1.15–13.79)	0.03

## Discussion

The oncologic outcomes after RP for patients with HRPCa disease merit specific attention. To date, there have only been a handful of studies where time to BCR and its effect on survival have been analyzed for patients with HRPCa treated with RP without neo- or adjuvant therapy (13–18). Indeed, most studies included mainly patients with favorable disease characteristics; only several of them focused especially on HRPCa. We managed to carry out a study that involved a huge number of 433 patients with HRPCa in comparison with other smaller studies (14–18).

In the presented cohort of men with pre-operative HRPCa features, the 5- and 10-year CPFS were 89.2 and 81%, and 5- and 10-year CSS were 95.6 and 90.1%, respectively. The strongest predictor for CP was time to BCR up to 1 year (HR: 2.7, *p* = 0.005). Early BCR (HR: 4.0, *p* = 0.03) together with LNI (HR: 4.1, *p* = 0.002) and R1 (HR: 4.2, *p* = 0.02) were detected as important predictors for CSS in the multivariate Cox regression analysis.

Several notable points should be mentioned analyzing the role of BCR and time to BCR regarding disease progression in the HRPCa population. To date, PSA remains the most important tool for following patients with PCa after curative treatment. Although surgical treatment provides good control of the disease, some of the patients might experience BCR. During the study period, almost half of men (47.8%) harbored BCR. Previous reports showed that although age was not a significant factor, other preoperative factors such as PSA, cT, biopsy GS, percentage of positive biopsy cores, number of high-risk factors according to D'Amico classification, pathological stage, GS, LNI, and surgical margin (SM) status were predictive factors for BCR (2, 8, 10, 12, 16, 22–27). Our results also proved the strength of all these risk factors for BCR even in the HRPCa population, but the strongest predictors when comparing preoperative factors were GS 8–10 and PSA value ≥ 20 ng/ml. Freedland et al. (22) showed that patients having preoperative PSA ≥ 20 ng/ml present a more than three times greater chance to have BCR compared to those patients who have preoperative PSA <6 ng/ml. We also managed to find that there is a 2-fold greater chance for BCR if the preoperative PSA is ≥20 ng/ml compared to PSA <10 ng/ml. Pathological GS 8–10, pT3b, and positive SM have the strongest impact on BCR analyzing post-operative parameters (from *p* = 0.014 to *p* = 0.005, Table 3).

Another important finding that should be noted is time to BCR. At the end of the study, 207 men (47.8%) harbored BCR, with the majority of 61% occurring within the first year after RP. These numbers are quite high compared to the recently published Chow et al. (28) study, where they had 44.6% of cases with early BCR. However, the authors presented fewer patients with preoperative high-risk features, which have a significant influence on the rate of BCR. Theoretically, PSA should be undetectable after RP within 21–30 days, considering that PSA's half-life period is 3.15 days (29). Unfortunately, some of the patients have persistent PSA that could have an impact on PCa progression. Lee et al. (17) noticed that if there is still residual prostatic tissue left after RP, which could be the case especially with HRPCa, PSA should be detectable afterwards. The question remains whether it is a residual benign prostatic tissue, extra prostatic tumor sites, or micro metastasis. The role of persistent PSA has been investigated recently by several investigators and ≥0.1 ng/ml PSA value was relied on as definition of PSA persistence (29–33). However, studies of this field are scant or focus on subgroups, such as LNI disease or patients with sRT. Only one very recent study has been aimed at looking for the possible effect of persistent PSA on the long-term oncological outcomes (32), but the real importance of PSA persistence needs to be clarified. Out of 433 men, 130 (30%) had PSA persistence in the cohort presented herein, which is similar to the Bianchi et al. series with LNI patients (32), but higher compared to Preisser or McDonald studies that had more favorable pathological features (31, 33). The population with persistent PSA is heterogeneous. Out of 130 patients, 57 (43.8%) had PSA between 0.1 and 0.2 ng/ml, and 44 of 57 (77.2%) harbored BCR during the study period. Only 11 of 57 (19.3%) had BCR within 1 year, which associates with lower risk for disease progression. Our sub-analysis shows that persistent PSA lost significance for prediction of disease progression in the multivariable regression when analyzed together with early BCR.

In the multivariable analysis, we identified several factors (higher pT and early BCR) that are associated with CP. However, the strongest factor was early BCR (*p* = 0.005). These data coincided with those in the studies of other authors (2, 13, 34, 35). Interestingly, our study identified correlation of LNI, R1, and pathological GS in the univariable, but they were not significant in multivariable analysis. Antonarakis et al. (35) presented a study where patients, after RP, never received adjuvant or salvage therapy before the development of CP. They also did not find correlation in the multivariable analysis between LNI, R1, and CP. However, they found correlation between high grades of pathological GS. Pound et al. (36) emphasize that 1 out of 3 patients with BCR will eventually have CP. In our study, CP was diagnosed in 23.7% (49/207) of cases with BCR, whereas Carver et al. (34) had only 14% of cases. It should be mentioned that Carver et al. included patients with more favorable disease characteristics. In our study, the majority of CP (38/49, 77.56%) appeared for patients having early BCR.

Time to BCR is also crucial for CRD as this has already been demonstrated in our multivariable analysis (*p* = 0.03). Similar results are found in other studies (12, 13, 37). Briganti et al. (13) appropriately point out correlation between early BCR and CRD. It should be mentioned that Briganti et al. defined early BCR as that which occurred in the first 3 years after RP. Pompe et al. (37) demonstrated that CRD correlates with early BCR (≤1 year), pathological GS, and short PSA-DT. We did not calculate PSA-DT and pathological GS was not a significant factor for CRD in our study. However, we present that LNI and R1 are significant for CRD, which contradicts the arguments found in the Pompe et al. study. Heterogeneous patient cohorts in the aforementioned studies did not allow the identification of a single predictor for PCa progression, but early BCR is one of them, especially in patients with high-risk features.

The limitations of our study include the use of a single institution. According to EAU guidelines (9), PLND should be recommended for all patients having HRPCa features. However, that was not the case in daily practice up to 2010, and in our study, PLND was done not for all HRPCa. We also did not calculate PSA-DT and did not include details of tumor size.

The strengths of our study include a high number of men with HRPCa features. We succeeded in collecting long follow-up data after RP. None of the patients received neo- or adjuvant treatment.

The results presented herein show that HRPCa patients are at high risk for BCR and time to BCR plays a very important role in the prediction of CP and CRD. For these patients, follow-up strategy should be personalized particularly in the first year after RP. Early BCR (up to 1 year) could be useful for counseling and decision making in the additional treatment setting.

## Data Availability Statement

The datasets generated for this study are available on request to the corresponding author.

## Author Contributions

ZV had full access to all the data in the study, takes responsibility for the integrity of the data, the accuracy of the data analysis, participated in data collection, data analysis, and manuscript writing. ZV and DM performed study design, data analysis, and manuscript writing. MJ contributed to manuscript writing.

### Conflict of Interest

The authors declare that the research was conducted in the absence of any commercial or financial relationships that could be construed as a potential conflict of interest.
