# Supplementary material for: Significance of Time Until PSA Recurrence After Radical Prostatectomy Without Neo- or Adjuvant Treatment to Clinical Progression and Cancer-Related Death in High-Risk Prostate Cancer Patients
Source: Front Oncol. 2019 Nov 22;9:1286. doi: 10.3389/fonc.2019.01286 (PMC6883747; doi:10.3389/fonc.2019.01286)

Supplementary Material

# Supplementary Figures and Tables

| Table S1. Multivariable logistic regression analyses predicting the presence of CP and CRD | | | | | |
| --- | --- | --- | --- | --- | --- |
|  | **CP**  **Multivariable analysis** | | **CRD**  **Multivariable analysis** | |  |
| Predictors | Odds ratio (95% CI) | p value | Odds ratio (95% CI) | p value |  |
| After RP GS  6  3+4 vs. 6  4+3 vs. 6  8 vs. 6  9-10 vs. 6 | Reference  0.27 (0.05-1.54)  0.26 (0.04-1.73)  0.72 (0.13-3.9)  2.16 (0.4-11.56) | 0.14  0.16  0.7  0.37 | - | - |  |
| pathological stage  T2  T3a vs. T2  ≥T3b vs. T2 | Reference  1.07 (0.3-3.79)  2.65 (0.74-9.52) | 0.91  0.13 | - | - |  |
| PSA persistence | 1.01 (0.45-2.54) | 0.89 | 1.5 (0.43-5.45) | 0.51 |  |
| LNI | 0.88 (0.45-1.74) | 0.71 | 3.1 (1.06-9.3) | 0.006 |  |
| R1 | 1.69 (0.78-3.64) | 0.18 | 4.2 (1.23-14.38) | 0.023 |  |
| Early vs Late BCR | 2.49 (1.06-5.88) | 0.037 | 3.2 (1.2-14.22) | 0.03 |  |
|  | | | | |  |

Figure S1. BPFS rate according to number of risk factors of D’Amico classification.

#
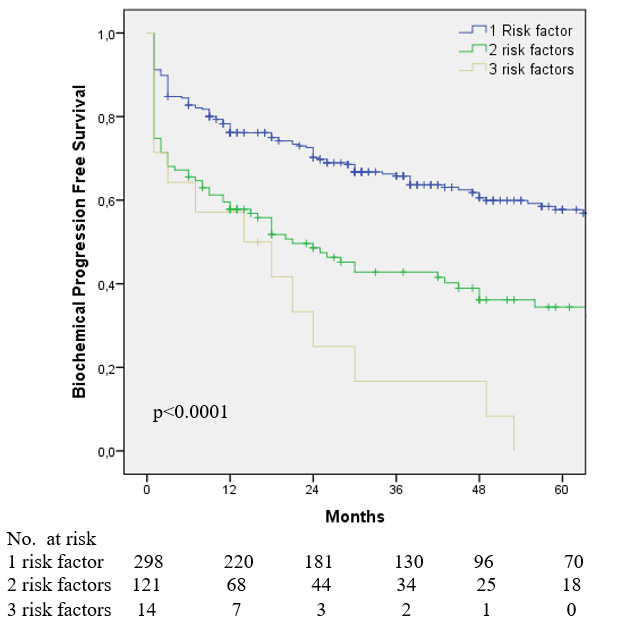

Supplement: Supplementary file 1 [file Table_1.DOCX]
